# Dietary starch to lipid ratios influence growth performance, nutrient utilisation and carcass traits in broiler chickens offered diets with different energy densities

**DOI:** 10.1371/journal.pone.0205272

**Published:** 2018-10-10

**Authors:** Ali Khoddami, Peter V. Chrystal, Peter H. Selle, Sonia Yun Liu

**Affiliations:** 1 Poultry Research Foundation, Sydney School of Veterinary Science, Faculty of Science, The University of Sydney, Camden NSW, Australia; 2 Sydney Institute of Agriculture, Faculty of Science, The University of Sydney, Sydney NSW, Australia; 3 Baiada Poultry Pty Limited, Pendle Hill, NSW, Australia; 4 School of Life and Environmental Sciences, Faculty of Science, The University of Sydney, Sydney NSW, Australia; INIA, SPAIN

## Abstract

Twelve experimental diets with three levels of energy densities (11.25, 12.38 and 13.50 MJ/kg) and fours levels of starch to lipid ratios (14:1, 12:1, 7:1, 4:1) were offered to 288 male Ross 308 broiler chickens. All the diets were formulated to contain consistent digestible lysine to metabolisable energy ratios (0.87 g digestible lysine/MJ AMEn) and ideal amino acid ratios. Growth performance was monitored from 7 to 27 days post-hatch and parameters of nutrient utilisation (AME, AMEn, AME:GE ratios, N retention) were determined from 24 to 26 days post-hatch. Apparent protein (N) and starch digestibility coefficients, carcass yield and composition were determined at 27 days post-hatch. There were no interactions between energy densities and starch to lipid ratios on growth performance and carcass weights (*P* > 0.05). Feed intake was reduced with increased energy densities (*P* < 0.001). Weight gain and FCR were improved with increased dietary energy densities (*P* < 0.0001). Starch to lipid ratios linearly increased weight gain (r = 0.448, *P* = 0.001) and feed intake (r = 0.509, *P* < 0.001) without influencing FCR (*P* > 0.75). Both nutrient densities and starch to lipid ratios significantly impacted on carcass weight and yield. Heavier carcass weights and higher yields were observed in broiler chickens offered diets with high nutrient density (*P* ≤ 0.001). Carcass weight (r = 0.441, *P* < 0.005) was positively correlated with starch to lipid ratios and this tended to be the case for carcass yield (r = 0.277, *P* = 0.057) too. However, there were interactions on lipid concentrations in carcass (*P* < 0.001) as broiler chickens offered diet containing the lowest nutrient density and the highest starch to lipid ratio had the highest lipid carcass concentration of 12.94%. In conclusion, protein and energy need to be considered in tandem in practical diet formulation, especially in diets containing high crystalline amino acid inclusions. The impact of lipid on feed intake and starch on carcass lipid concentrations should also be taken into consideration.

## Introduction

The influence of protein and energy on growth performance and carcass traits in broiler chickens have been intensively studied in the literature and the investigation is ongoing because the performance of modern broiler chickens improve every couple of years as a result of successfully selective breeding program. However, starch and lipid are the two major source of energy in typical broiler diets and little information about the influence of starch and lipid on bird performance and nutrient utilisation has gained in the literature. Starch is the most abundant macronutrient in broiler diets and its primary function is to provide energy for maintenance and growth and escalating dietary starch concentrations would increase lipid concentrations in carcass. Dietary lipid for poultry, including poultry fat and vegetable source oil, has higher energy density but is more expensive than starch. Its concentration is also critical to pellet quality and feed intake. Liu et al. [1] reported a significant 8.8% reduction in feed intake in birds offered diets containing higher lipid concentrations and such responses are often seen in practice. This could be due to dietary lipid negatively impacting on pellet quality [2], and/or delaying gastric emptying [3]. In the present study, the influence of dietary starch to lipid ratios on growth performance, carcass compositions and nutrient utilization was investigated.

Amino acids and energy are considered in tandem in the formulation of practical diets for monogastric animals [4] and Lewis [5] suggested grams per unit of energy may be the best method of expressing amino acid requirements when animals are given *ad libitum* access of feed. Indeed, poultry nutritionists may choose to fix the ratio of protein and energy in practical least-cost feed formulation in order to conveniently change dietary nutrient densities according to the fluctuation of feed ingredient prices. This approach is suitable when broiler chickens tend to eat to a constant energy intake regardless of the energy density of the diet [5]; however, it remains debatable whether modern broilers eat to constant energy intake [6, 7]. Liu et al. [1] reported quadratic relationship between protein to energy ratios and growth performance in broiler chickens. Similarly, Gous et al. [7] reported broken-stick response to protein to energy ratios and efficiency of protein utilisation. Therefore, in order to avoid introducing confounding factors into the present study, all the experimental diets were formulated to consistent ratios of digestible lysine to apparent metabolisable energy (0.87 g digestible lysine/MJ nitrogen–corrected apparent metabolisable energy); however, digestible lysine concentrations were ranged from 9.5 to 12.1 g/kg. The hypothesis was that increasing nutrient density would enhance feed conversion efficiency and the starch to lipid ratio would influence the carcass composition of broiler chickens.

## Materials and methods

The present study is one of a series to investigate the influence of macronutrients on feed intake, weight gain and carcass composition in broiler chickens. The details of bird management, diet preparation and sample collection were similar to methodology described in Liu et al. [1].

### Diets preparation

The feeding study comprised twelve dietary treatments with three energy density levels (11.25, 12.38 and 13.50 MJ/kg) and four starch to lipid ratios (14, 12, 7, 4) as shown in Table 1. All diets were formulated to contain similar digestible lysine to metabolisable energy (0.87 g digestible lysine/MJ nitrogen–corrected apparent metabolisable energy) and ideal amino acid ratios. The analysed dietary starch concentrations ranged from 276 to 414 g/kg and lipid ranged from 26 to 76 g/kg. There were variations between formulated and analysed macronutrient concentrations as shown in Table 1. The composition and calculated nutrient specifications in experimental diets 1–4 and diets 9–12 for broiler chickens are shown in Table 2. Diets 5–8 contained intermediate levels as they were derived from 50–50 blends of the corresponding low and high nutrient density diets.

**Table 1:** Formulated and analysed macronutrient concentrations (g/kg) in experimental diets.

	Formulated				Analysed^1^			
Diet	AMEn^2^	Protein	Lipid	Starch	Protein	Lipid	Starch	Dry matter (DM)
1	11.25	166	21	420	184	27	359	862
2	11.24	185	25	373	204	26	335	859
3	11.26	190	35	330	198	39	306	861
4	11.25	186	63	280	188	73	276	875
5	12.37	181	23	468	194	26	389	858
6	12.37	193	30	427	203	31	374	858
7	12.37	193	51	368	202	52	350	855
8	12.38	199	71	316	202	76	296	867
9	13.50	195	26	515	214	29	414	854
10	13.50	200	34	480	221	36	404	856
11	13.49	195	67	407	205	63	376	846
12	13.51	213	79	352	219	75	314	854

^1^protein was analysed as N x 6.25; lipid was analysed by Soxhelt extraction

^2^nitrogen corrected apparent metabolisable energy, MJ/kg

**Table 2:** Diet composition and calculated nutrient specifications in diets 1–4 and diets 9–12 for broiler chickens from 7–27 days post-hatch.

Ingredients (g/kg)	Diet 1	Diet 2	Diet 3	Diet 4	Diet 9	Diet 10	Diet 11	Diet 12
Oats	0	25	50	355	0	0	0	0
Maize	451	444	353	138	617	648	540	458
Wheat mill run	0	63	147	0	80.5	2.9	0	0
Maize starch	119	27	0	0	0	0	0	0
Peas	43	120	200	200	132	179	180	180
Soybean meal	214	230	151	83	0	0	143	194
Canola meal	0	0	45	150	0	0	0	28
Casein	19	0	0	0	112	106	35	35
Soybean oil	0	0	8	29	0	7	43	56
Lysine HCl	0.5	0	0.1	0.2	0.3	0	2.1	0
Methionine	2.2	1.9	1.8	1.5	3.6	3.5	3.8	3.1
Threonine	0.6	0.2	0.2	0.2	1.7	1.4	2.0	0.8
Tryptophan	0.0	0.0	0.0	0.0	0.1	0.1	0.2	0.0
Valine	0.0	0.0	0.0	0.0	0.0	0.0	1.2	0.0
Arginine	0.0	0.0	0.0	0.0	5.3	4.5	1.9	0.0
Isoleucine	0.0	0.0	0.0	0.0	0.7	0.5	1.3	0.1
Salt	2.0	2.0	2.0	1.8	2.0	2.0	1.3	2.0
Sodium bicarbonate	1.8	1.2	0.7	0.9	1.3	1.0	2.0	1.0
Limestone	9.5	9.8	9.9	8.2	10.3	10.2	9.6	9.4
Dicalcium phosphate	12.0	11.0	9.0	10.0	11.0	11.0	11.0	10.0
Phytase^1^	0.1	0.1	0.1	0.1	0.1	0.1	0.1	0.1
Choline chloride 60%	0.4	0.4	0.4	0.4	0.4	0.4	0.4	0.4
Vitamin-mineral premix^2^	2.0	2.0	2.0	2.0	2.0	2.0	2.0	2.0
Cellulose	103	43	0	0	0	0	0	0
Celite	20	20	20	20	20	20	20	20
AMEn (MJ/kg)	11.25	11.24	11.26	11.25	13.5	13.5	13.49	13.51
Protein	166	185	190	186	195	200	195	213
Lipid	21	25	35	63	26	34	67	79
Starch	420	373	330	280	515	480	407	352
Calcium	8.7	8.8	8.7	8.8	8.7	8.7	8.6	8.6
Fibre	22.8	35.2	49.3	73.3	26.8	30.4	31.4	33.5
Total phosphorus	5.0	5.7	6.1	5.9	4.5	4.7	5.1	5.3
Available P	4.4	4.4	4.3	4.4	4.3	4.4	4.3	4.3
Lysine^3^	9.5	9.5	9.5	9.5	12.1	12.1	12.1	12.1
Methionine	4.7	4.3	4.2	4.1	7.2	7.1	6.6	6.2
Methionine+cysteine	7.0	7.0	7.0	7.0	9.0	9.0	9.0	9.0
Threonine	6.3	6.3	6.3	6.3	8.1	8.0	8.2	8.0
Tyrosine	1.8	2.0	2.0	1.9	2.0	2.0	2.0	2.2
Isoleucine	6.7	7.0	6.7	6.6	8.5	8.5	8.5	8.5
Leucine	13.8	14.3	11.4	12.8	17.4	17.7	15.4	16.9
Arginine	9.9	11.9	12.2	12.1	12.6	12.6	12.6	12.6
Valine	7.6	7.8	7.8	8.0	10.1	10.2	9.7	9.7
Histidine	4.1	4.5	4.5	4.3	4.2	4.3	4.4	5.1
Sodium	1.8	1.8	1.8	1.8	1.8	1.8	1.8	1.8
Potassium	7.0	8.7	8.8	7.4	3.9	4.4	7.0	8.0
Chloride	2.0	2.0	2.0	2.0	2.0	2.0	2.0	2.0

^1^Axtra PHY TPT was included at the rate of 1000 FTU/kg

^2^The vitamin-mineral premix supplied per tonne of feed: [MIU] retinol 12, cholecalciferol 5, [g] tocopherol 50, menadione 3, thiamine 3, riboflavin 9, pyridoxine 5, cobalamin 0.025, niacin 50, pantothenate 18, folate 2, biotin 0.2, copper 20, iron 40, manganese 110, cobalt 0.25, iodine 1, molybdenum 2, zinc 90, selenium 0.3

^3^Digestible amino acids

Maize was hammer-milled through 6.0 mm screen prior to mixing with the other ingredients and diets were cold-pelleted and then crumbled. Acid insoluble ash (Celite^TM^ World Minerals, Lompoc, CA, USA) was included in the diets at 20 g/kg as an inert marker to determine nutrient digestibility coefficients at the distal jejunum and distal ileum at 27 days post-hatch. Starch concentration were determined by a procedure based on dimethyl sulfoxide, α-amylase and amyloglucosidase, as described in Mahasukhonthachat et al. [8]. Nitrogen concentrations were determined as outlined by Siriwan et al. [9]. Lipid concentration was determined by using the automated Soxhlet extraction as described in Luque de Castro and Priego-Capote [10].

### Bird management

This feeding study was approved by the Animal Ethics Committee of the University of Sydney (Project No. 601). Male, one day-old chicks (Ross 308) were received from a commercial hatchery and were offered a commercial starter diet to 7 days post-hatch. They were then individually identified (wing-tags), weighed and allocated into bioassay cages, with dimensions of 750 mm in width, 750 mm in length and 510 mm in height, on the basis of body weight in an environmentally-controlled facility. There was no statistical difference on the average body weight for each cage at the beginning of the feeding study. Each of the dietary treatment was offered to four replicate cages (6 birds per cage) or a total of 288 chicks from 7 to 27 days post-hatch. Broilers had unlimited access to water and feed under a ‘23-hour-on-1-hour-off’ lighting regime for the first three days and then under a ‘16-hour-on-8-hour-off’ lighting regime for the remainder of the study. Room temperature was maintained at 32°C for the first week, then gradually decreased to 22 ± 1°C by the end of the third week and maintained at the same temperature until the end of the feeding study. Body weight and feed intake were recorded weekly from which feed conversion ratios (FCR) were calculated. The incidence of dead or culled birds was recorded daily and their body-weight was used to adjust FCR calculations.

### Sample collection and chemical analysis

Total excreta were collected from 24–26 days post-hatch from each cage to determine parameters of nutrient utilisation which included apparent metabolisable energy (AME), metabolisable energy to gross energy (GE) ratios (AME:GE), nitrogen retention and N-corrected apparent metabolisable energy (AMEn). Excreta were air-forced oven dried for 24 h at 80°C. The GE of diets and excreta were determined by bomb calorimetry using an adiabatic calorimeter (Parr 1281 bomb calorimeter, Parr Instruments Co., Moline, IL).

The jejunum is reported to be the major site of glucose and amino acid absorption but the extent of nutrient digestion at the end of ileum, which is often expressed as apparent ileal digestibility coefficient, is usually reported in the literature [11, 12]. Apparent digestibility coefficients of starch and protein were determined in both distal jejunum and distal ileum in the present study. At day 28, all birds were euthanized by intravenous injection of sodium pentobarbitone and the small intestine was removed and digesta samples were collected in their entirety from the distal jejunum and distal ileum. The jejunum was demarcated by the end of the duodenal loop and Meckel’s diverticulum and the ileum by Meckel’s diverticulum and the ileo-caecal junction. Digesta was taken from the segment posterior to the respective mid-points. Digesta samples from birds within a cage were pooled, homogenized, freeze-dried and ground through 0.5mm screen. Then, the samples were analysed for the content of starch and protein as previously described.

Two birds from each cage whose body weight was close to the cage mean were selected for carcass composition analyses. The carcass was weighed with feathers but without viscera to calculate carcass yield before processing. Then, the carcass was autoclaved, ground and freeze-dried to analyse for nitrogen, lipid, DM concentrations and GE as described previously.

### Calculations

The AME values were calculated on a DM basis from the following equation:

$${AME}_{diet}=\frac{\left(Feed\;intake\times {GE}_{diet})-(Excreta\;output\times {GE}_{excreta}\right)}{\left(Feed\;intake\right)}$$

AME:GE Ratios were calculated by dividing AME by the GE of the appropriate diets. N contents of diets and excreta were determined using a nitrogen determinator (Leco Corporation, St Joseph, MI) and N retentions calculated from the following equation:

$$Retention\;(\%)=\frac{\left(Feed\;intake\times {Nutrient}_{diet})-(Excreta\;output\times {Nutrient}_{excreta}\right)}{\left(Feed\;intake\times {Nutrient}_{diet}\right)}\times 100$$

N-corrected AME (AMEn MJ/kg DM basis) values were calculated by correcting N retention to zero using the factor of 36.54 kJ/g N retained in the body [13]. Apparent metabolisable energy intakes (MJ/day DM) were calculated from dietary energy densities and average daily feed intakes over the entire feeding period.

Acid insoluble ash (AIA) was included in the diets at 20 g/kg as an inert marker. Apparent digestibility coefficients of starch and protein (N) were calculated by the following equation:

$$Digestibility\;Coefficient=\frac{{\left(Nutrient/AIA\right)}_{diet}-{(Nutrient/AIA)}_{digesta}}{{\left(Nutrient/AIA\right)}_{diet}}$$

Starch and protein (N) disappearance rates (g/bird/day) were deduced from feed intakes over the final phase of the feeding period from the following equation:

$$\begin{array}{l} Nutrient\;disappearance\;rate\\ \;={Feed\;intake\;}_{g/bird}\times {Dietary\;nutrient}_{g/kg}\\ \;\times Appartent\;digetsibility\;coefficient \end{array}$$

### Statistical analysis

Experimental data were analysed using JMP 9.0.0 (SAS Institute Inc. JMP Software. Cary, NC) and response surfaces were generated with R 3.1.3 software. The experimental units were replicate cage means and statistical procedures included analyses of variance using the general linear models and a probability level of less than 5% was considered to be statistically significant by Student’s t-test. Additionally, the response surface plots were constructed so that the effects from changing factor levels on the examined responses can be visualized and they were generated by generalized additive model with thin plate regression splines as the smoothing function, the details of analysis and interpretation of the contour plots can be reviewed in Solon-Biet et al. [14]. Linear and quadratic regressions were also considered in statistical analyses of the experimental data. Pairwise correlation was conducted to explore the relationships between apparent digestibility coefficients, nutrient utilisations, growth performance and carcass traits and a probability level of less than 5% was considered to be statistically significant.

## Results

The mortality rate during the experimental period was 1.7% and it was not related to dietary treatments (*P* > 0.30). The average weight gain and FCR for all the experimental treatments from 7 to 27 days post-hatch was 1591 g/bird and 1.407, respectively, which were clearly superior to the 2014 Ross 308 performance objectives of 1292 g/bird weight gain with and FCR of 1.463. The influence of nutrient densities and starch to lipid ratios on growth performance and carcass traits from 7–27 days post-hatch is shown in Table 3 (S1 Table). There were no interactions between energy densities and starch to lipid ratios on growth performance and carcass weight and yield (*P* > 0.05). Feed intake was reduced by 6.4% with increased energy densities (2314 versus 2167 g/bird, *P* < 0.001) and weight gain and FCR were improved with increased dietary energy densities by 10.5% (1511 versus 1670 g/bird) and 15.3% (1.533 versus 1.298), respectively (*P* < 0.001). There were significant linear relationships between starch to lipid ratios with weight gain (r = 0.448, *P* = 0.001) and feed intake (r = 0.509, *P* < 0.001). Broiler chickens offered diets with the lowest starch to lipid ratio had significantly inferior weight gain (1498 g/bird), feed intake (2098 g/bird) and carcass weight (1505 g/bird) in comparison to birds offered diets containing the other three levels of starch to lipid ratios. In contrast, FCR was not influenced by dietary starch to lipid ratios (*P* = 0.794). Both nutrient densities and starch to lipid ratios had significant impacts on carcass weight and yield. Carcass weight (r = 0.441, *P* = 0.002) and yield (r = 0.277, *P* = 0.057) decreased with reduced starch to lipid ratios. Broiler chickens offered diets with the highest starch to lipid ratio had significantly higher carcass yield compared to the other treatment groups (*P* = 0.001). There were treatment interactions on carcass GE (*P* = 0.002). In low density diets, broiler chickens offered starch: lipid ratio of 14 had significantly higher carcass GE than birds offered diets containing the other three starch: lipid ratios. Differently, in medium density diets, starch: lipid ratios did not influence carcass GE. In high density diets, broiler chickens offered starch: lipid ratio of 4 had significantly lower carcass GE than birds offered diets containing the other three starch: lipid ratios. There were no dietary effects on carcass protein concentrations (*P* > 0.50) and DM concentrations (*P* > 0.15). There were interactions on carcass lipid concentrations (*P* < 0.001). Broiler chickens offered diet containing the lowest nutrient density and highest starch to lipid ratio had the most carcass lipid (12.94%) whereas broiler chickens offered diet containing the highest nutrient density and lowest starch to lipid ratio had the least carcass lipid (8.25%).

**Table 3:** The influence of nutrient densities and starch to lipid ratios on performance and carcass traits in broiler chickens from 7–27 days post-hatch.

Treatment	Nutrient density	Starch:lipid ratios	Weight gain (g/bird)	Feed intake (g/bird)	FCR (g/g)	Carcass weight (g/bird)	Carcass yield (%)	Carcass composition			
								GE (MJ/kg DM)	Protein (% as-is)	Lipid^1^ (% as-is)	DM (% as-is)
1	Low	14	1645	2504	1.522	1601	90.0	27.3^a^	22.77	12.94^a^	39.68
2	Low	12	1539	2344	1.523	1564	89.7	24.9^c^^d^	22.68	9.13^c^^d^	39.09
3	Low	7	1510	2311	1.531	1545	89.9	25.4^b^^c^^d^	22.42	9.72^b^^c^^d^	38.51
4	Low	4	1348	2098	1.556	1390	89.6	25.6^b^^c^	22.48	10.25^b^^c^	39.48
5	Medium	14	1655	2306	1.393	1752	90.9	25.8^b^^c^	22.75	10.38^b^^c^	39.06
6	Medium	12	1621	2255	1.390	1612	89.4	25.3^b^^c^^d^	22.99	9.92^b^^c^	40.20
7	Medium	7	1603	2205	1.374	1585	90.2	25.4^b^^c^^d^	22.70	9.89^b^^c^^d^	39.19
8	Medium	4	1491	2083	1.398	1501	89.5	25.9^b^^c^	22.07	10.33^b^^c^	38.39
9	High	14	1672	2220	1.328	1673	91.0	25.6^b^^c^	22.84	10.19^b^^c^	39.49
10	High	12	1672	2155	1.292	1738	90.7	26.1^b^	22.77	11.28^b^	40.24
11	High	7	1683	2179	1.295	1723	90.1	26.1^b^	22.48	11.33^a^^b^	40.36
12	High	4	1654	2113	1.279	1623	90.7	24.4^d^	22.56	8.25^c^^d^	38.96
		SEM	39.73	62.74	0.0208	44.4	0.26	0.38	0.341	0.574	0.519
*Main effect*: Nutrient density											
Low			1511^c^	2314^a^	1.533^a^	1525^c^	89.8^b^	25.8	22.59	10.51	39.19
Medium			1593^b^	2212^b^	1.389^b^	1613^b^	90.0^b^	25.6	22.63	10.13	39.21
High			1670^a^	2167^b^	1.298^c^	1689^a^	90.6^a^	25.6	22.66	10.26	39.76
Starch:lipid ratios											
14			1658^a^	2343^a^	1.414	1675^a^	90.6^a^	26.2	22.79	11.17	39.41
12			1610^a^	2251^a^^b^	1.402	1638^a^	90.0^b^	25.4	22.81	10.11	39.84
7			1599^a^	2232^b^	1.400	1618^a^	90.1^b^	25.7	22.54	10.31	39.35
4			1498^b^	2098^c^	1.411	1505^b^	89.9^b^	25.3	22.37	9.61	38.94
*P-value*											
Nutrient density			<0.001	<0.001	<0.0001	<0.0001	0.001	0.580	0.952	0.645	0.224
Starch:lipid ratio			<0.0001	0.007	0.794	<0.001	0.013	0.032	0.342	0.018	0.230
Interactions			0.074	0.406	0.609	0.175	0.083	0.002	0.918	<0.001	0.181

^abcd^ Means within a column not sharing common superscripts are significantly different

^1^Lipid concentration in carcass was calculated from equation developed in the Poultry Research Foundation, University of Sydney: (3.9683×GE-75.622) × Dry matter

The influence of energy densities and starch to lipid ratios on distal jejunal and distal ileal apparent digestibility coefficients and disappearance rates of starch and protein are shown in Table 4 (S1 Table). Escalating energy densities increased protein (N) digestibilities in the distal jejunum (*P* <0.001) and distal ileum (*P* < 0.0001). Broiler chickens offered high density diets had significantly higher apparent protein digestibility coefficients in the distal jejunum and distal ileum than broiler chickens offered low and medium density diets. Boiler chickens offered diets containing starch: lipid ratio of 4 had significantly lower apparent protein digestibility coefficient in the distal ileum (*P* = 0.004) in comparison to birds offered diets with all the other three ratios. Dietary nutrient density did not influence apparent starch digestibility coefficients in the distal jejunum (*P* = 0.497) and distal ileum (*P* = 0.639). Starch to lipid ratios significantly influenced apparent starch digestibility coefficient in the distal jejunum (*P* = 0.023) and the only significant difference was observed between birds offered diets with starch: lipid ratio of 14 and 12 (0.967 versus 0.934). There was an interaction between nutrient densities and starch to lipid ratios on apparent digestibility coefficients of starch in the distal ileum (*P* < 0.001). In low and medium density diets, broiler chickens offered diets containing starch: lipid ratio of 7 had significantly lower apparent starch digestibility coefficient in the distal ileum in comparison to birds offered diets with all the other three ratios. However, in high density diets, broiler chickens offered diets containing starch: lipid ratio of 4 had significantly lower apparent ileal starch digestibility coefficient compared to birds offered diets with all the other three ratios.

**Table 4:** The influence of nutrient densities and starch to lipid ratios on apparent digestibility coefficients of starch and protein (N) in distal jejunum and distal ileum at 27 days post-hatch.

Treatment	Nutrient density	Starch:lipid ratios	Protein (N) digestibilities		Starch digestibilities		Protein (N) disappearance rate (g/bird/day)		Starch disappearance rate (g/bird/day)	
			Jejunum	Ileum	Jejunum	Ileum	Jejunum	Ileum	Jejunum	Ileum
1	Low	14	0.719	0.844	0.957	0.981^a^	16.2	19.0	42.2	43.0
2	Low	12	0.700	0.839	0.920	0.974^a^^b^	16.8	20.1	36.1	38.2
3	Low	7	0.683	0.829	0.961	0.941^d^	15.1	18.3	32.7	32.9
4	Low	4	0.730	0.813	0.953	0.977^a^	14.4	16.0	27.6	28.3
5	Medium	14	0.757	0.871	0.971	0.985^a^	16.9	19.5	43.5	44.2
6	Medium	12	0.749	0.854	0.927	0.978^a^	17.2	19.5	39.2	41.3
7	Medium	7	0.722	0.838	0.948	0.965^b^^c^	16.1	18.6	36.6	37.3
8	Medium	4	0.724	0.831	0.943	0.977^a^	15.2	17.5	29.1	30.1
9	High	14	0.774	0.874	0.973	0.980^a^	18.3	20.6	44.4	44.8
10	High	12	0.796	0.875	0.954	0.979^a^	18.9	20.8	41.5	42.6
11	High	7	0.782	0.875	0.959	0.983^a^	17.5	19.5	39.3	40.3
12	High	4	0.792	0.867	0.941	0.960^c^	18.3	20.1	31.2	31.9
		SEM	0.0218	0.0086	0.013	0.0043	0.70	0.65	1.30	1.30
*Main effect*: Nutrient density										
Low			0.708^b^	0.831^c^	0.946	0.969	15.6^b^	18.4^b^	34.6^c^	35.6^b^
Medium			0.738^b^	0.848^b^	0.947	0.976	16.3^b^	18.8^b^	37.1^b^	38.2^a^
High			0.786^a^	0.873^a^	0.956	0.976	18.3^a^	20.3^a^	39.1^a^	39.9^a^
Starch:lipid ratios										
14			0.750	0.863^a^	0.967^a^	0.972	17.1^a^^b^	17.9^c^	43.4^a^	44.0^a^
12			0.748	0.856^a^	0.934^b^	0.977	17.6^a^	18.8^b^^c^	38.9^b^	40.7^b^
7			0.729	0.847^a^^b^	0.956^a^^b^	0.963	16.2^b^^c^	20.1^a^	36.2^c^	36.8^c^
4			0.749	0.837^b^	0.946^a^^b^	0.972	16.0^c^	19.7^a^^b^	29.3^d^	30.1^d^
*P-value*										
Nutrient density			<0.001	<0.0001	0.497	0.639	<0.0001	<0.001	<0.001	0.001
Starch:lipid ratio			0.721	0.004	0.023	0.037	0.014	<0.001	<0.0001	<0.0001
Interactions			0.702	0.367	0.553	<0.001	0.718	0.175	0.853	0.789

^abcd^ Means within a column not sharing common superscripts are significantly different

Disappearance rates of starch and protein in the distal jejunum and distal ileum were influenced by nutrient densities (*P* < 0.001) and starch to lipid ratios (*P* < 0.02). Generally, disappearance rates of starch and protein were improved with increased nutrient densities regardless of the site of small intestine. There were positive correlations between starch to lipid ratios and protein (N) disappearance rate in the distal jejunum (r = 0.306, *P* = 0.034) and distal ileum (r = 0.580, *P* < 0.0001); starch disappearance rate in the distal jejunum (r = 0.354, *P* = 0.015) and distal ileum (r = 0.542, *P* < 0.0001).

The influence of nutrient densities and starch to lipid ratios on nutrient utilisation in broiler chickens is shown in Table 5 (S1 Table). There were treatment interactions between nutrient densities and starch to lipid ratios for AME, AME:GE ratios and AMEn (*P* < 0.005). Starch: lipid ratios did not influence AME in broiler chickens offered low density diets; whereas, in high density diets, birds offered starch: lipid ratios of 4 and 7 significantly higher AME than broiler chickens offered diets with the other two ratios. Broiler chickens offered the lowest nutrient density diet and the lowest starch to lipid ratio had the lowest AME (13.11 MJ/kg, *P* < 0.0001) and AMEn (12.28 MJ/kg, *P* < 0.0001). N retention reduced with starch to lipid ratios (r = 0.474, *P* = 0.001) and the lowest N retention (68.2%) was observed when the starch to lipid ratio was equal to 4 (*P* = 0.012). Increasing energy densities from 11.25 to 13.50 MJ/kg significantly reduced water intake by 14.6% (378 versus 323 ml/day/bird, *P* = 0.004).

**Table 5:** The influence of nutrient densities and starch to lipid ratios on nutrient utilisations in broiler chickens from 24–26 days post-hatch.

	Nutrient	Starch:lipid	AME^1^	AME:GE	AMEn	N retention	Excreta moisture	Water intake (ml per day per bird)
Treatment	density	ratios	(MJ/kg)		(MJ/kg)	(%)	(%)	
1	Low	14	13.59^e^	0.741^f^	12.57^e^^f^	71.9	67.0	379
2	Low	12	13.67^e^	0.740^f^	12.73^e^^f^	69.5	67.4	378
3	Low	7	13.69^e^	0.724^f^	12.82^e^	65.9	67.5	395
4	Low	4	13.11^e^	0.687^g^	12.28^f^	68.0	61.8	360
5	Medium	14	14.62^b^^c^^d^	0.777^d^^e^	13.63^c^^d^	71.8	63.8	330
6	Medium	12	16.44^a^	0.850^a^	14.99^a^	72.3	70.6	352
7	Medium	7	14.48^c^^d^	0.755^e^^f^	13.65^c^^d^	69.3	64.6	324
8	Medium	4	15.13^b^	0.749^e^^f^	14.30^b^	68.6	66.6	355
9	High	14	14.33^d^	0.777^d^^e^	13.45^d^	70.8	65.1	314
10	High	12	14.93^b^^c^	0.830^a^^b^	14.02^b^^c^	72.2	65.7	292
11	High	7	16.03^a^	0.813^b^^c^	15.07^a^	72.2	70.2	349
12	High	4	15.85^a^	0.792^c^^d^	14.97^a^	68.0	66.6	336
		SEM	0.132	0.0110	0.131	1.38	1.84	21.5
*Main effect*: Nutrient density								
Low			13.52	0.723	12.60	68.8	65.9	378^a^
Medium			15.17	0.783	14.14	70.5	66.4	340^b^
High			15.28	0.803	14.42	70.8	66.9	323^b^
Starch:lipid ratios								
14			14.18	0.765	13.22	71.5^a^	65.3	341
12			15.01	0.806	13.91	71.3^a^^b^	67.9	341
7			14.73	0.764	13.85	69.2^b^^c^	67.4	356
4			14.70	0.742	13.85	68.2^c^	65.0	350
*P-value*								
Nutrient density			<0.0001	<0.0001	<0.0001	0.081	0.737	0.004
Starch:lipid ratio			<0.001	<0.0001	<0.0001	0.012	0.152	0.777
Interactions			<0.0001	0.002	<0.0001	0.144	0.065	0.468

^abcdefg^ Means within a column not sharing common superscripts are significantly different

^1^AME, apparent metabolisable energy; AME:GE, ratio of AME and GE in the diets; AMEn, nitrogen corrected AME

## Discussions

### Macronutrients and feed intake

**Fig 1:**
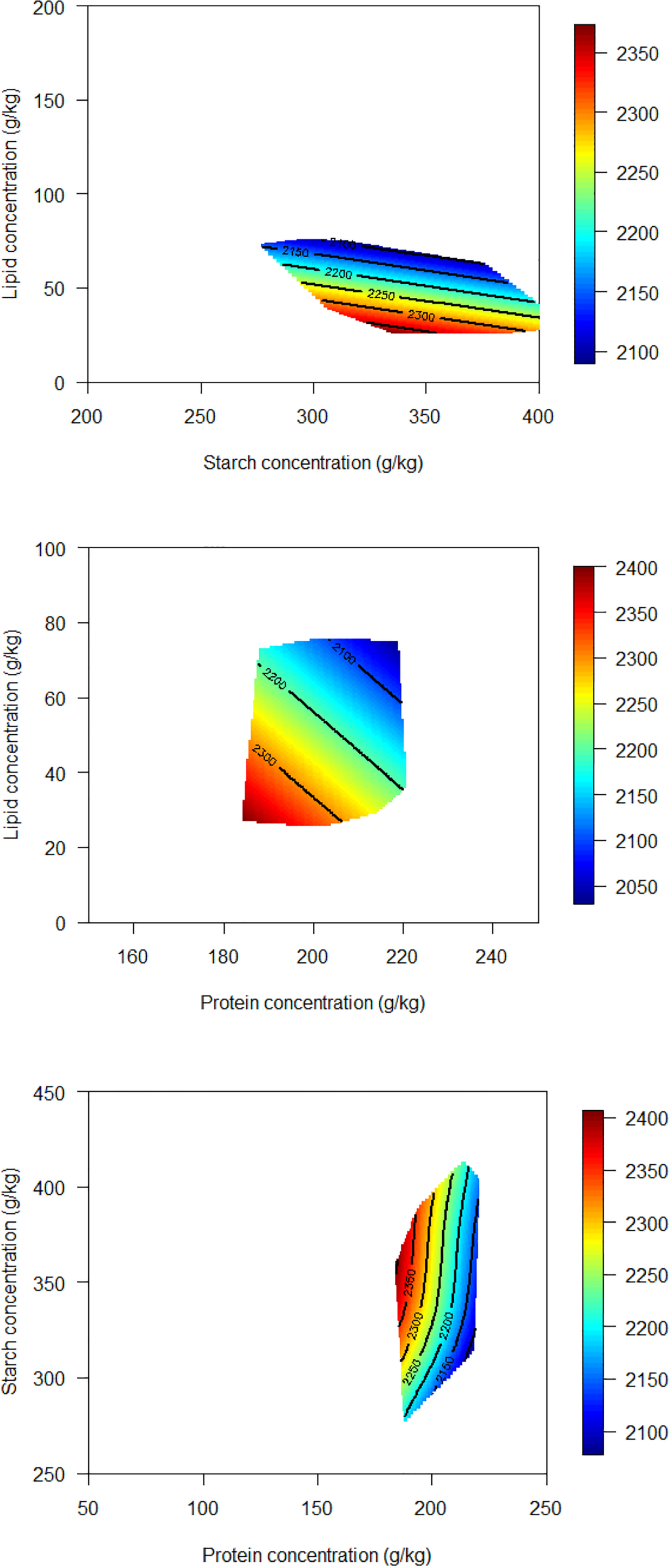
The influence of analysed dietary starch, protein and lipid concentrations on feed intake in broiler chickens from 7–27 days post-hatch.

The response surface plots were constructed so that the effects from changing factor levels on the examined responses can be visualized and they were generated by generalized additive model with thin plate regression splines as the smoothing function. The influence of analysed dietary starch, protein and lipid concentrations on feed intake in broiler chickens from 7–27 days post-hatch is shown in Fig 1. Both lipid and protein are more influential on feed intake in poultry than dietary starch concentrations [1, 15]. When dietary lipid and starch concentrations were compared, contours were almost parallel to the x-axis and this indicates that dietary lipid concentration had a greater impact on feed intake than starch and feed intake was reduced with increased lipid concentrations. This observation is consistent with previous findings [1, 15]. Response surface graphs generated in Liu et al. [15] showed consistent patterns and when lipid was compared with starch and protein, contours were almost parallel to x-axis which suggested that feed intake decreased with increased lipid concentration in broiler diets. Subsequently, Liu et al. [1] showed that increasing dietary lipid concentration from 40 to 75 g/kg generated an 8.8% reduction in feed intake (1999 versus 1823 g/bird, *P* < 0.0001), a 15.9% reduction in weight gain (1526 versus 1284 g/bird, *P* < 0.0001) and FCR was compromised by 13.8% FCR (1.360 versus 1.547, *P* < 0.0001). The lipid-induced triggering of the ‘ileal brake’ may be the cause of feed intake reductions in diets with higher lipid concentrations. Martinez et al. [3] reported that the intraluminal infusion of lipids modulates gastrointestinal motility and delays gastric emptying by decreasing the frequency of the gastric cycle, increasing duodenogastric refluxes, and elongating the migrating myoelectric complex. Subsequently, Maljaars et al. [16] proposed that perfusion of fat into the ileum inhibits small intestinal digesta transit, gastric emptying, pancreaticobiliary and gastric acid secretion and food intake in mammals. Dietary factors influencing lipid utilisation in poultry include the degree of saturation of fatty acids, the inclusion of lipid, the positional distribution of fatty acids within the glyceride molecule, the feed grain on which the diet is based, dietary calcium levels and feed processing [17]. Consistently, Maljaars et al. [16] concluded that the factors influencing inhibitory effects of lipid on gastric emptying which include fatty acid or triacylglycerol, chain length of fatty acids, degree of saturation of fatty acids and quantity of lipid. In humans, a dose-dependent decrease in pancreatic and biliary secretion was observed in response to doses of lipid of 0, 50 or 100 mg/min perfused into the ileum [18]. Therefore, it is possible that higher lipid intake triggered the ‘ileal brake’ and reduced feed intake. Another possible reason for reduced feed intakes with higher dietary lipid concentration could be the inferior pellet quality. It is recognised in practice that dietary lipid is required to maintain the throughput of feed-mill; however, high inclusion of lipid, especially vegetable oils, may compromise pellet hardness and quality. Kleyn [2] suggested a non-linear formulation approach for diets with high lipid levels because dietary energy densities may not increase with dietary lipid concentrations in a linear manner.

With the dietary protein and starch concentration comparisons in Fig 1, the contours almost parallel the y-axis and this indicates dietary protein concentration had a more pronounced impact on feed intake than starch and feed intakes were reduced with increased protein concentrations. Suthama et al. [19] found that broiler chickens offered diets containing high protein concentrations (306 g/kg) had their feed intakes significantly reduced by 26.9% (588 versus 804 g/bird) in comparison to birds offered a low protein (201 g/kg) but iso-energetic diet from 15 to 27 day post-hatch. Liu et al. [1] reported quadratic effects of dietary protein concentration on feed intake in broiler chickens from 7–28 days post-hatch. When crude protein concentration was less than 200 g/kg, feed intake increased with increasing dietary protein concentrations; whereas, when crude protein concentration was higher than 240 g/kg, feed intake decreased with increasing dietary protein concentrations. The authors suggested that a balance between protein and non-protein energy was required for optimal growth performance in broiler chickens. In the present study, the dietary digestible lysine to energy ratios were fixed across all the experimental diets; however, non-protein energy was derived from either starch or lipid. Both lipid and protein had more pronounced impact on feed intake than starch (Fig 1).

### Protein intake target

**Fig 2:**
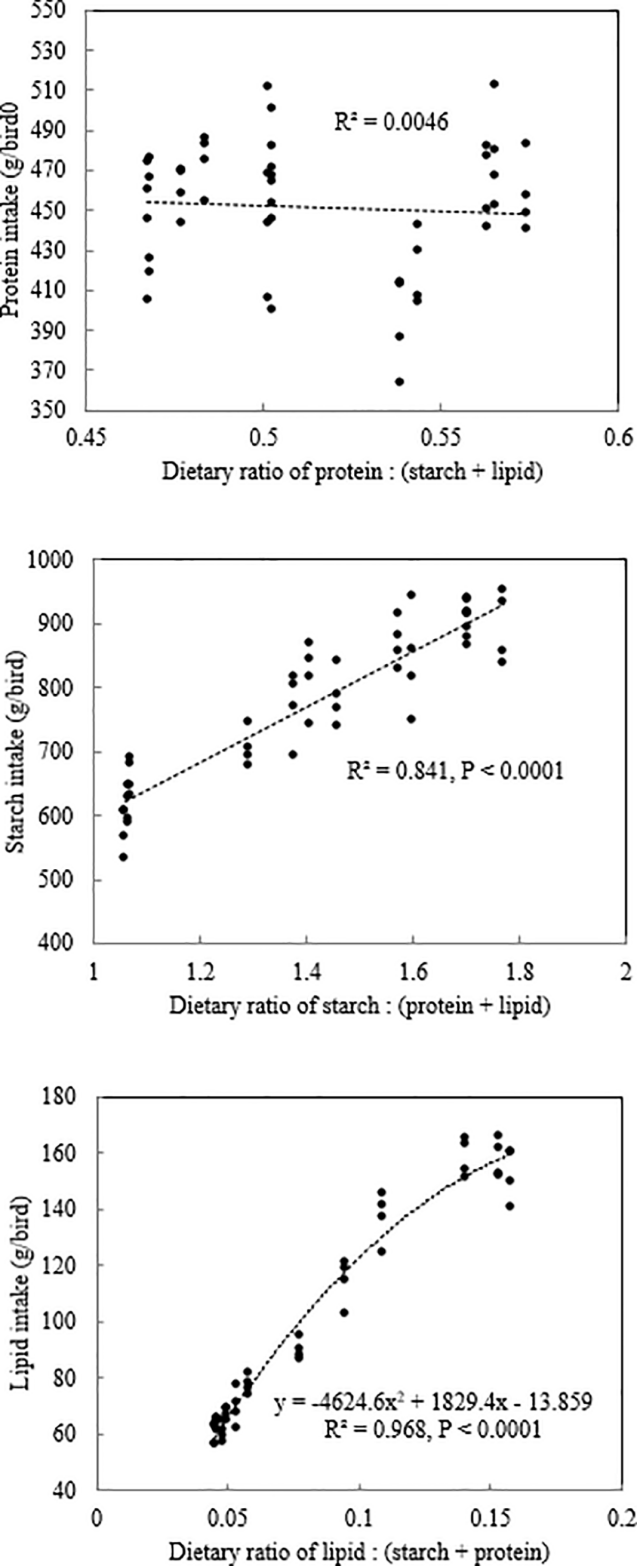
The relationships between nutrient to non-nutrient ratios and their corresponding nutrient intake from 7–27 days post-hatch.

Simpson and Raubenheimer [20] advanced the protein leverage hypothesis and suggested humans and animals prioritised protein intake when they were forced to exchange protein intake against that of carbohydrate and lipid derived from nutritionally unbalanced diets. This means they tended to maintain a constant protein intake relative to starch and lipid intakes. The relationship between protein intake from 7 to 27 days post-hatch and protein to non-protein ratios in diets in the present study is shown in Fig 2. Protein intake maintained constant when the ratio of dietary protein and non-protein concentrations increased (R^2^ = 0.005) and this suggests that broiler chickens had a constant protein intake of approximately 450 g/bird during the experimental period regardless of the dietary protein proportions. In contrast, starch intakes increased linearly (R^2^ = 0.841, *P* < 0.0001) with its proportion in the diets. When the ratio of starch to non-starch dietary components increased, there was also an increase in the intake of starch. There was a quadratic relationship between lipid intake and ratios of lipid to non-lipid components. Lipid intake increased with the ratio of lipid to non-lipid components in the diets and it reached maximum of 167 g/bird when the ratio of lipid to non-lipid components equaled 0.198. This suggests that broiler chickens do not eat to constant starch and lipid intake target; however, that lipid intake reached a plateau may be due to the impact of lipid on total feed intake in broiler chickens. Increasing dietary lipid concentrations from 23.5 to 71.0 g/kg or decreasing starch to lipid ratios significantly depressed feed intake by 10.5% (2343 versus 2098 g/bird, *P* = 0.007). This observation accords with the findings of Liu et al. [1] as they reported an 8.8% reduction in feed intake (1823 versus 1999 g/bird; *P* < 0.0001) in broiler chickens offered diets containing the higher lipid concentration. Consistently, high lipid diets in the present study contained higher fibre concentrations (mean values: 24.8 versus 53.4 g/kg) as shown in Table 2. Therefore, it appears that higher fibre concentrations in high lipid diets had negative impacts on feed intake in broiler chickens. Another possible explanation for the reduction of feed intake is the lipid-induced triggering of the ‘ileal brake’ as suggested in Martinez et al. [3]. These researchers found that intraluminal infusion of lipids in poultry modulates gastrointestinal motility including an increase in duodenogastric refluxes or episodes of reverse peristalsis. The researchers suggested that these actions could delay gastric emptying and increase transit time, which is consistent with the “ileal brake” mechanism similar to that described in mammals.

### Protein and energy balance

It is straightforward that increased nutrient density improved feed conversion and energy utilisation (Table 5). A further possibility is the higher inclusions of crystalline amino acids in the high nutrient density diets. On average, the low nutrient density diets (1–4) contained 2.35 g/kg crystalline amino acids; whereas, the high nutrient density diets (9–12) contained 9.55 g/kg crystalline amino acids. In addition to lysine, methionine and threonine, high nutrient density diets were also supplemented with tryptophan, valine, arginine and isoleucine. Melnick et al. [21] suggested that all essential amino acids must be liberated from protein at appropriate rates to optimise protein utilisation. Goldberg and Guggenheim [22] determined concentrations of lysine, methionine and tryptophan in the portal blood stream after feeding rats with different protein sources. Ten minutes after feeding casein, the lysine concentration of 153 μg/ml exceeded the lysine concentration of 71 μg/ml from overheated soy flour by more than a two-fold factor. Thus the rate of protein digestion, absorption and transition of amino acids into the portal blood stream from ‘intact’ proteins can vary enormously. Crystalline or synthetic amino acids do not require digestion and are rapidly absorbed [23]. This was perhaps reflected in Table 4 as broiler chickens offered high density diets had significantly higher protein digestibilities and protein disappearance rates. On average, diet 9, 10 and 11 contained 11.4 g/kg synthetic amino acids which represented 5.8% of total dietary protein. Consequently, broiler chickens offered high nutrient density diets had significantly higher apparent protein digestibilities in the jejunum and ileum (*P* < 0.001).

Table 6 summaries the findings that feed intake was influenced by the balance of protein and energy and once the balance was achieved feed conversion efficiency may be improved. When the ratio between protein and energy was held constant, increasing nutrient density increased weight gain, reduced feed intake and improved feed conversion efficiency. When dietary protein was constant, increasing energy may increase or depress feed intake depending on the protein concentration in the diet. For example, Liu et al. [24] offered broiler chickens three diets to select from and all three diets contained 17.5 g/kg digestible lysine and 300 g/kg crude protein but different energy levels (11.04, 12.58 and 14.12 MJ/kg). Broiler chickens strongly preferred diets containing the highest energy to balance the high protein concentration and achieved an FCR of 1.217 compared to the average FCR of 1.473 from 10 to 31 days post-hatch. However, Gous et al. [25] reported reductions in feed intake when either dietary protein or energy were increased while the other was held constant when broiler chickens were offered only one diet at one time. This is consistent with the protein intake target theory discussed above and, broiler chickens consumed to constant protein intake and feed intake would be depressed once this target is attained.

**Table 6:** The summary of how dietary protein (digestible lysine), energy and their ratios influence feed intake and FCR.

Digestible lysine	ME	Ratio	Feed intake	FCR
↑	↑	Constant	↓	↓
Constant	↑	↓	↑↓	↓
↑	Constant	↑	↑↓	↓

### Carcass composition

In the present study, weight gain and FCR were correlated (*P* < 0.05) with apparent digestibility coefficients of protein in the distal jejunum and distal ileum (Table 7). Increasing protein digestibilities in the distal jejunum and distal ileum increased weight gain and improved feed conversion efficiency. Consistently, weight gain and FCR were correlated (*P* < 0.05) with energy utilisation (AME, AME:GE and AMEn). There were no treatment effects on protein concentrations in carcass (*P* > 0.30). The balance between protein and energy is required for muscle protein deposition [12] and the lack of treatment response on carcass protein concentrations could be due to the fixed ratio of protein to metabolisable energy in the diets. Amino acid and glucose are both required for muscle protein deposition and a surplus of glucose may be deposit as body lipid [12]. On average, increasing dietary starch to lipid ratio from 4 to 14 increased lipid concentration in carcass from 9.61 to 11.17% (*P* = 0.018).

**Table 7:** Pairwise correlations between performance parameters and digestibilities and nutrient utilisations in broiler chickens.

		Apparent nitrogen digestibility		Nutrient utilisations			
Response		Distal jejunum	Distal ileum	AME	AME : GE	N retention	AMEn
Weight gain	r =	0.258	0.586	0.476	0.551	0.311	0.449
	P =	0.047	<0.0001	<0.001	<0.0001	0.017	<0.001
FCR	r =	-0.503	-0.587	-0.418	-0.333	0.025	-0.644
	P =	<0.0001	<0.0001	0.001	0.010	0.850	<0.0001
Carcass weight	r =	0.285	0.523	0.332	0.403	0.180	0.331
	P =	0.028	<0.0001	0.010	0.002	0.174	0.011
Carcass yield	r =	0.387	0.526	0.045	0.139	-0.028	0.086
	P =	0.002	<0.0001	0.738	0.294	0.835	0.519

## Conclusions

In the present study, increasing nutrient density increased weight gain, decreased feed intake and improved feed conversion efficiency in broiler chickens from 7 to 27 days post-hatch. Lipid had a more pronounced impact on feed intake than dietary starch concentrations and as predicted in Fig 1, increasing dietary lipid concentrations from 25 to 75 g/kg depressed feed intake from 2350 to 2100 g/bird. Broiler chickens tended to consume to a constant protein intake because protein intake did not change with the protein proportions in the experimental diets. A surplus of energy derived from starch may increase carcass lipid concentrations and the balance between protein and energy is pivotal for optimal feed conversion and body protein deposition. Protein and energy need to be considered in tandem in practical diet formulation, as diets containing high crystalline amino acid inclusions with low crude protein contents may require a lesser energy expenditure along the digestive tract. The impact of lipid on pellet durability and feed intake and the impact of starch on carcass lipid concentrations should also be taken into consideration.

## Supporting information

S1 Table Individual cage raw data. (XLSX)
